# Phytopathogenic Fungi and Toxicity

**DOI:** 10.3390/toxins13100689

**Published:** 2021-09-28

**Authors:** Maria Michela Salvatore, Anna Andolfi

**Affiliations:** 1 Department of Chemical Sciences, University of Naples ‘Federico II’, via Cinthia, 80126 Naples, Italy; mariamichela.salvatore@unina.it; 2 Institute for Sustainable Plant Protection, National Research Council, 80055 Naples, Italy; 3 BAT Center—Interuniversity Center for Studies on Bioinspired Agro-Environmental Technology, University of Naples ‘Federico II’, 80055 Naples, Italy

Phytopathogen fungi are responsible for serious plant diseases which might negatively affect crop productivity [1]. Some of these fungi are also documented as opportunist human pathogens that can cause infections in immunocompromised individuals [2]. In this respect, fungal interaction with other organisms is of great interest since fungi employ an array of biochemical and mechanical strategies to infect their host in order to access nutrients. During infection, polymer-degrading enzymes or secondary metabolites are produced as virulence factors [1]. Furthermore, fungi produce mycotoxins on crops, and this represents a considerable risk to human and animal health [3].

In addition, phytopathogenic fungi have also been studied as biocontrol agents against pests [4,5] or for their ability to produce compounds with a wide variety of biological activity (including herbicidal, antibiotic, and antifungal activities) [6,7].

Studies of phytopathogenic fungi might be interesting in order to understand the mechanism of fungal pathogenicity and virulence, to develop strategies for screening of disease, and for the application of natural compounds with bioactivities.

This special issue is focused on fungal pathogenicity and virulence in order to develop strategies for screening of disease and for the application of natural compounds with bioactivities. Several studies have examined the production of toxic metabolites by fungi involved in plant diseases and a couple of review papers give good overviews on aspects linked to the production of metabolites.

The paper by Singh et al. [8] investigated the effect of growth conditions on the production of aflatoxins by two morphologically similar species that co-exist in West Africa, *Aspergillus aflatoxiformans* and *Aspergillus minisclerotigenes*. The assay designed in this work for differentiating two species could be useful for identifying specific etiologic agents of aflatoxin contamination episodes in West Africa and other regions where the two species are sympatric, especially when phylogenetic analyses based on multiple gene segments are not practical. A newly HRAM UPLC-MS/MS method for the screening of the phytotoxic metabolites botrydial and dihydrobotrydial associated with the phytopathogenic fungi *Botrytis cinerea* was developed and validated by Huang et al. [9]. Results presented in this work suggest that curcumin-mediated photosensitization is effective in the control of *B. cinerea* spoilage.

The diversity and toxigenic potential of fungal species causing fruitlet core rot (FCR) disease in pineapple were investigated by Barral et al. [10]. In particular, *Fusarium ananatum* and *Talaromyces stollii* are the most spread fungi responsible of FCR and mycotoxins, such as fumonisin B1, fumonisin B2 and beauvericin, were found in infected pineapple fruitlets. In vitro, phenolic acid amendment reduces mycotoxin accumulation.

*Neofusicoccum parvum* is a fungal pathogen infecting a wide range of plant hosts. Pour et al. [11] investigate the genome of *N. parvum* leading to the identification of six putative genes encoding necrosis and ethylene-inducing proteins (NLPs).

*Pyrenophora* is another fungal genus responsible for a number of plant diseases. Moolhuijzen et al. [12] conducted the first comparative analysis of biosynthetic gene cluster across three majors fungal pathgens in the *Pyrenophora* genus.

The paper by Fragola et al. [13] provided preliminary datasets on the airborne Viridiplantae and fungi communities, and on potential pathogenic fungi and plant-derived aeroallergens for a coastal site of the Central Mediterranean.

Lastly, two review papers within the special issue deal with the occurrence of secondary metabolites produced by plant pathogenic fungi. In this respect the review by Urbaniak et al. [14] highlight the extensive variability of *Fusarium* cyclodepsipeptide mycotoxins by describing the chemistry, biosynthesis, and occurrence of beauvericins, enniatins, and beauvenniatins in foods and feeds.

In the review paper of Salvatore et al. [15] the available data concerning the detection, bioactivities, and occurrence of secondary metabolite produced by *Lasiodiplodia theobromae* were compiled. In this review, the wide variety of studied *L. theobromae* strains allows for evaluating the differences in metabolite production that might be related to different hosts, as well as to the symbiotic association established between a fungus and host. One hundred and thirty-four chemically defined compounds belonging to the classes of secondary metabolites and fatty acids have been reported from over 30 *L. theobromae* isolates. Compounds reported include cyclohexenes and cyclohexenones, indoles, jasmonates, lactones, melleins, phenols, and others. Most of the existing bioactivity studies of *L. theobromae* metabolites have assessed their potential phytotoxic, cytotoxic, and antimicrobial activities.

## Data Availability

Not applicable.
